# Inter-rater Agreement in Multi-informant Reports of Psychosocial Functioning of Pediatric Brain and Solid Tumor Survivors

**DOI:** 10.1007/s10880-024-10059-9

**Published:** 2024-12-04

**Authors:** Manali Zope, Matthew C. Hocking

**Affiliations:** 1 The Children’s Hospital of Philadelphia, Philadelphia, PA USA; 2 Perelman School of Medicine, University of Pennsylvania , Philadelphia, PA USA

**Keywords:** Pediatric cancer, Multi-informant psychosocial assessment, Multi-informant behavioral assessment, Inter-rater reliability, Quality of life

## Abstract

Objectives: For pediatric cancer survivors in the post-treatment, school-reintegration period, integrating multi-informant reports and promoting a mutual clinician–family–school understanding of the child’s needs are critical for comprehensive care. This study evaluated patterns of agreement between child, parent, and teacher reports of psychosocial functioning in pediatric brain tumor survivors (PBTS) and non-CNS solid tumor survivors (PSTS). Participants and Methods: PBTS (*n* = 51) and PSTS (*n* = 34) age 7–14 who received tumor-directed therapy completed the study. Parents and teachers completed the CBCL/TRF and SSIS, and parents and children completed the PedsQL and PROMIS peer relationships. Intra-class correlation coefficients, % disagreements, t-tests, and correlations quantified inter-rater agreement. Results: Analysis yielded poor-to-moderate ICC levels across measures. Parent–teacher agreement was higher for reports of externalizing symptoms. Parents had higher ratings of child-internalizing problems, but lower ratings of overall social skills than teacher ratings. Parents had higher ratings of child emotional functioning and social skills compared to self-reports. Conclusions: Findings underscore the necessity for integrating multi-informant reports of psychosocial functioning in assessment for pediatric cancer survivors. Findings also highlight critical gaps in mutual parent–teacher–child understanding, indicating the need for increased collaboration in the post-treatment period.

**Supplementary Information:**

The online version contains supplementary material available at 10.1007/s10880-024-10059-9.

## Introduction

Pediatric brain tumor survivors (PBTS) and non-central nervous system solid tumor survivors (PSTS) are vulnerable to behavioral and psychosocial difficulties. Intensive treatment protocols lead to increased school absenteeism during critical developmental periods, which inhibit child psychosocial development (Hocking et al., 2017; Tsimicalis et al., 2017). Compared to typically developing children, survivors are at greater risk for issues with anxiety, depression, post-traumatic stress, and emotional instability, as well as hyperactivity and attention problems (Bessell, 2001; Brinkman et al., 2016, 2018; Peterson & Jacobson, 2021). Additionally, studies incorporating teacher and peer reports show that survivors experience greater social adjustment problems including greater social isolation and lower social acceptance (Efe et al., 2022; Willard et al., 2015).

Given this increased prevalence of behavioral and psychosocial challenges, the Standards for Psychosocial Care for Children with Cancer advocate for consistent collaboration in clinical assessment and intervention support between clinicians, families, and schools, especially in the post-treatment school-reintegration period (Thompson et al., 2015). Incorporating multiple informants in clinical assessment enables insight into environment-specific child functioning patterns and differences in informant perceptions, both of which may be influenced by prolonged treatment and school absenteeism. Thus, integrating ratings from multiple informants across environments is critical for comprehensive behavioral and psychosocial assessment in the post-treatment period to accurately identify those who would benefit from intervention. Further, a mutual clinician–family–school understanding of the child’s needs can ensure appropriate individualized support during school reintegration (Thompson et al., 2015).

Research on multi-informant assessment highlights the prevalence of low-moderate agreement across informants’ ratings of child behavioral and psychosocial functioning (Achenbach et al., 1987; De Los Reyes & Kazdin, 2005). For instance, studies in typically developing children suggest low-moderate agreement between parents and teachers, wherein parents report higher levels of problem behaviors (Ende & Verhulst, 2005). Parents and teachers generally have higher agreement on ratings of externalizing problems compared to internalizing problems due to their higher observability (De Los Reyes & Kazdin, 2005). Parent–child agreement on psychosocial functioning and HrQoL is also low moderate, with the directionality of ratings being population dependent. Parents of children in cancer or chronically ill groups tend to rate functioning lower than child reports (Parsons et al., 1999). In addition, the level of agreement between parents and children on measures of HrQoL seems to decrease as the clinical severity and perceived consequences of the child’s physical and psychological functioning increases (Papp et al., 2022; Radicke et al., 2021). These findings of low-moderate levels of agreement indicate distinct perceptions of child functioning and highlight critical gaps in mutual understanding across informants of the child’s behavior and psychosocial functioning.

Although these discrepancies may complicate the clinical process and conceptualization of a child’s functioning, the established psychometric properties of measures support the use of multiple informants’ perspectives, and discrepancies offer unique clinical utility. The level of agreement between informants may offer insight into the extent of mutual understanding of the child’s functioning and needs, wherein domains with low agreement indicate critical gaps in shared understanding. The directionality of informant agreement (e.g., lower parent rating than teacher) may offer valuable insight into context-specific child behaviors and further inform where gaps in communication may arise. In addition, agreement patterns between parent, teacher, and child reports have clinical utility as studies have found that the level of agreement directly predicts the occurrence of psychological and physiological problems and inversely predicts youth intervention responses’ long term (De Los Reyes & Epkins, 2023; Ferdinand et al., 2004).

Despite their vulnerability to psychosocial difficulties, research on patterns of agreement for ratings of psychosocial functioning in PBTS and PSTS is lacking. Investigating agreement patterns for PBTS and PSTS is important to inform clinical practices that support the child's behavioral and psychosocial functioning in the critical post-treatment reintegration period. Thus, the primary purpose of this study was to characterize the level and directionality of agreement between child, parent, and teacher reports of psychosocial functioning in PBTS and PSTS. Given the current evidence of low-moderate agreement in pediatric populations, it was hypothesized that there would be an overall pattern of low inter-rater agreement across ratings of psychosocial functioning in PBTS and PSTS. Similar to observed patterns in other pediatric populations, it was also hypothesized that parents would have lower ratings of child psychosocial functioning compared to teachers and self-report.

## Methods

### Sample

Participants were survivors of either a brain tumor (*n* = 49) or a non-CNS solid tumor (*n* = 34) between the ages of 7 and 14 who received tumor-directed therapy (surgery, chemotherapy, and/or radiation therapy) and were English speaking. Individuals were excluded on the following criteria: having a multi-system genetic condition that impacts cognitive functioning (e.g., neurofibromatosis type 1, trisomy 21), having a cognitive or developmental delay prior to the tumor diagnosis, or (for non-CNS solid tumors only) receiving treatments that affect the CNS (e.g., total body irradiation). Caregiver inclusion criteria included being English speaking, living with the child at least 50% of the time, and having actively participated in the child’s treatment. Teachers were identified by the caregiver and completed questionnaires remotely. Out of the 127 families that were approached about the study (*n* = 72 PBTS, 55 PSTS), 83 (65.4%; *n* = 49 PBTS, 34 PSTS) agreed to participate. From the overall sample of 83 participants, 65 teachers completed the SSIS, and 67 teachers completed the TRF. Consenting and non-consenting participants did not differ in demographic or medical characteristics. See Table 1 for participant demographic and clinical characteristics.

**Table 1:** Participant demographic and medical variables

Variables	*n* (%) or *M* ± SD
Age in years	10.98 ± 2.26
IQ	101.37 ± 13.36
Female sex	34 (39.5%)
Race	
Caucasian	59 (71.1%)
African American	12 (14.5%)
Asian	7 (8.4%)
Multi-Ethnic	2 (2.4%)
Unreported	3 (3.6%)
Hispanic/latinx	
Hispanic/latinx	6 (7.2%)
Caregiver education	
High school or less	16 (19.3%)
Some college	16 (19.2%)
At least college graduate	51 (61.4%)
Household income	
Less than 20 k	5 (5.95%)
20–49 K	19 (22.62%)
50–100 K	21 (25%)
100–124 K	15 (17.86%)
125 K +	20 (23.81%)
Unreported	4 (4.76%)
Age at tumor diagnosis in months	9.67 ± 3.19
Time since diagnosis in months	20.34 ± 28.26
Time since treatment completion in months	3.42 ± 1.73
Tumor types	
Brain tumor	49 (59.0%)
Solid tumor	34 (41.0%)
Treatment	
Surgery only	25 (30.1%)
Radiation only	2 (2.4%)
Chemo only	12 (14.5%)
Surgery + chemo	11 (13.3%)
Surgery + radiation	7 (8.4%)
Chemo + radiation	9 (10.8%)
All Three	15 (18.1%)
Unreported	2 (2.4%)
Time school missed in months	
Less than 2 months	36 (42.9%)
2–5 months	21 (25.0%)
5–8 months	14 (16.7%)
8–12 months	9 (10.7%)
More than one year	3 (3.6%)
Unreported	1 (1.2%)

### Procedure

The current study is a secondary analysis of baseline data collected as part of a larger longitudinal study conducted at a large, urban pediatric medical center (Albee et al., 2022; Hocking et al., 2020). Potentially eligible children were identified through tumor registries and cooperation with medical teams and contacted for recruitment through letters, phone calls, and in person clinic visits. Data used for analysis in this investigation were collected within 6 months of the child’s completion of tumor-directed treatment (*M* = 3.4 months, SD = 1.7 months). Relevant clinical information was extracted from the child’s medical chart, and children, parents, and teachers completed measures rating the child’s psychosocial functioning.

### Measures

The *Achenbach System of Empirically Based Assessment (ASEBA) Child Behavior Checklist (CBCL) and CBCL Teacher Report Form (TRF)* are two parallel broadband measures of child emotional and behavioral symptoms (Achenbach, 2001). The CBCL and TRF are composed of eight syndrome scale scores, six DSM-oriented scale scores, competence and adaptive scale scores, and broad band internalizing and externalizing problem scale scores. The items on both the CBCL and TRF are rated by caregivers and teachers on a 3-point Likert-type scale: Not True (0), Somewhat, or Sometimes True (1), or Often True (2), with T-scores between 65 and 69 indicating borderline problem severity, and T-scores 70 + indicating clinical problem severity. See Supplemental Table 1for the list of the psychosocial CBCL/TRF scales that are analyzed in this investigation with their corresponding internal consistencies (Cronbach’s alpha) for this sample and example items.

The *Social Skills Improvement System (SSIS)* is a broadband social competence assessment that was developed to screen social behavior difficulties and plan related interventions (Gresham & Elliot, 2008). The SSIS (parent and teacher form) includes items related to the child’s social skills and problem behaviors that are rated on a 4-point scale ranging from “never” to “almost always.” The social skills subdomain includes seven subscales (Cooperation, Communication, Assertion, Responsibility, Empathy, Engagement, and Self-Control) and the problem behaviors’ subdomain includes five subscales (Externalizing, Bullying, Hyperactivity–Inattention, Internalizing, Autism Spectrum) which are all calculated from norms and reported in raw scores (subscales) and standard scores (total social and behavior). See Supplemental Table 1 for the list of the psychosocial SSIS scales that are analyzed in this investigation with their corresponding internal consistencies (Cronbach’s alpha) for this sample and example items.

The *Pediatric Quality-of-Life Inventory Generic Core Scales’ Version 4.0 (PedsQL, ages 5–7 and 8–12)* is a 23-item measure that assesses a child’s and parents’ perceptions of health-related QoL designed specifically for populations of healthy and chronically ill children (Varni et al., 2001). The PedsQL is composed of four subscales: physical functioning, emotional functioning, social functioning, and academic functioning, with the latter three combining to form the psychosocial functioning composite score and all four combining to form the total score. Items are rated on a 5-point scale (ages 8–12) ranging from “never” to “almost always” and on a 3-point scale (self-report only, ages 5–7) using faces ranging from “not at all” to “a lot,” with higher scores indicating better functioning. The generic module was implemented instead of the cancer module in the larger study in order to enable comparisons with healthy and other chronically ill populations. See Supplemental Table 1 for the list of the psychosocial PedsQL scales that are analyzed in this investigation with their corresponding internal consistencies (Cronbach’s alpha) for this sample and example items.

The *Patient-Reported Outcomes Measurement Information System Peer Relationship Short Form (PROMIS-PR)* is a brief parent- and child-report measure assessing the child’s peer relationships quality, including participation and satisfaction in social activities and sociability (ability to get along with peers) (DeWalt et al., 2013). Items are framed “In the past 7 days…” with a 5-point response scale ranging from “never” to “almost always.” Higher T-scores indicate better perceptions of the child’s peer relationship quality. See Supplemental Table 1 for the PROMIS parent and self-report internal consistencies (Cronbach’s alpha) for this sample and example items.

### Analysis

Pediatric brain and non-CNS solid tumor survivor groups were combined for analysis to increase statistical power and the generalizability of the study to broader pediatric cancer populations. Analyses were conducted using SPSS Version 28.0. Descriptive statistics summarized participant demographic and medical variables. The level of inter-rater agreement on each CBCL/TRF, SSIS, PedsQL, and PROMIS subscale of interest was quantified and delineated using a two-way mixed, absolute agreement model of the Intra-class Correlation Coefficient (ICC). The inter-rater ICC is a proportion of the true variance (inter-child differences) of a multi-rater measure to the total variance (inter-child + inter-rater differences), which quantifies the extent to which the measure distinguishes between participants with diverging scores. Values of ICC range from 0 to 1, with values closer to 1 indicating higher inter-rater agreement. Agreement levels are delineated as follows: *ICC* < *0.5* = *poor*, *ICC* = *0.5 – 0.75* = *moderate*, *ICC* = *0.75 – 0.90* = *good*, and *ICC* = *0.9* ± *excellent* (Koo & Li, 2016*)*.

Patterns of agreement directionality (significantly higher/lower group ratings on a given subscale) were determined using t-tests between mean rater scores for each subscale. Pearson correlation coefficients evaluated the strength of linear associations between rater groups.

The percent of cases that are in significant disagreement for each subscale are determined by solving for the minimum distance of two significantly different scores using the Reliable Change Index (RCI) equation. Inter-rater ICC values calculated in this analysis were used for the reliability term, (${r}_{xx}$), in the RCI equation. Pairs with ratings at distances greater than or equal to the derived significant difference point were qualified as in disagreement. In addition, pairs with diverging clinical classifications (any level of impairment vs average and above) were qualified as in disagreement.

## Results

### Participants

A total of 83 children (39.5% female, on average 11 years old) and their parents were included in this investigation. Out of the sample of 83, a total of 66 teachers completed the SSIS and 67 teachers completed the TRF. Participant demographic and medical treatment information are included in Table 1.

### Quantifying Level of Agreement

**Fig. 1:**
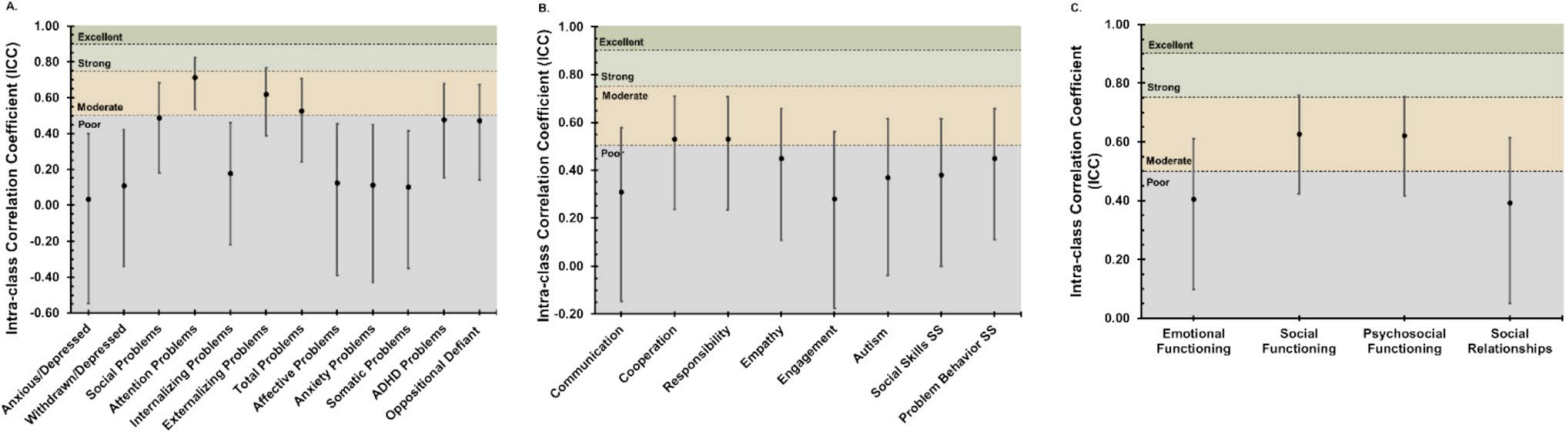
Comparison of inter-rater reliability across measures and subscales. Intra-class correlation coefficients (ICCs, represented as dots) and corresponding confidence intervals at *α* = 0.05 (CIs, represented as error bars) for parent–teacher and parent–child ratings. **A** Parent–teacher agreement on CBCL/TRF. **B** Parent–teacher agreement on SSIS. Raw scores were used for all subscales except standard scores for social skills and problem behavior composite scales. **C** Parent–child agreement on PedsQL and PROMIS

Figure 1A and Supplemental Table 2 summarize the ICC values for each analyzed CBCL/TRF scale, highlighting an overall pattern of low-to-moderate agreement between parents and teachers. Moderate ICC values were found only for the attention (ICC = 0.71, 95% CI = 0.54, 0.82), externalizing (ICC = 0.62, 95% CI = 0.39, 0.77), and total (ICC = 0.53, 95% CI = 0.24, 0.71) problem scales. Poor agreement (ICC < 0.5) was found on anxious/depressed, withdrawn/depressed, social problems, and internalizing problems’ syndrome scales and all five analyzed DSM scales (affective, anxiety, somatic, ADHD, oppositional).

Figure 1B and Supplemental Table 2 display the ICC values for the given SSIS scales, showing an overall pattern of poor agreement between parents’ and teacher’s ratings of child social functioning. The social skills’ standard score (ICC = 0.34, 95% CI = 0, 0.62) and problem behavior standard score (ICC = 0.45, 95% CI = 0.11, 0.66) both resulted in poor ICC values. Subscale raw score reliabilities were poor to moderate, ranging from Engagement (ICC = 0.28, 95% CI = − 0.18, 0.56) to Cooperation (ICC = 0.53, 95% CI = 0.24, 0.71) and Responsibility (ICC = 0.53, 95% CI = 0.23, 0.71).

Figure 1C and Supplemental Table 2 show the results of parent–child ICC reliability analysis on the PedsQL and PROMIS measures. Moderate ICC values (with CIs ranging poor–strong) were only found for the social functioning (ICC = 0.63, 95% CI = 0.42, 0.76) and psychosocial functioning (ICC = 0.62, 95% CI = 0.42, 0.75) PedsQL scales. The PedsQL emotional functioning scale (ICC = 0.41, 95% CI = 0.10, 0.61) and PROMIS peer relationships form (ICC = 0.39, 95% CI = 0.05, 0.61) resulted in poor ICC-derived reliabilities.

### Directionality and Magnitude of Agreement

*CBCL/TRF:* Significant inter-rater correlations between parents and teachers were seen on the following scales: Social, Attention, Externalizing, Total, ADHD, and Oppositional Defiant (*p’s* < 0.01, See Table 2). Mean parent ratings were significantly higher than teacher ratings on the Social and Total problem scales (*p’s* < 0.05) and the Withdrawn/Depressed, Internalizing, and Somatic problem scales (*p’s* < 0.01). Magnitudes between ratings (|parent – teacher|) range from Attention (2.81 ± 4.03) to Internalizing (12.10 ± 7.51).

**Table 2:** Inter-rater differences, magnitudes, and correlations

Measure/subscale		Mean rater 1 score (parent)	Mean rater 2 score (teacher/self)	Paired *T*-test* t*-statistic	Mean magnitude (|P–T/S|)	Pearson correlation
CBCL	Anxious/depressed	55.10 (7.11)	53.31 (5.06)	1.69	5.82 (6.61)	0.018
	Withdrawn/depressed	56.34 (7.52)	52.49 (5.46)	3.51**	6.27 (7.47)	0.069
	Social problems	55 (6.66)	53.01 (4.76)	2.44*	4.79 (5.00)	0.357**
	Attention problems	53.49 (5.42)	52.45 (4.83)	1.78	2.81 (4.02)	0.566**
	Internalizing problems	54.34 (10.25)	47.79 (8.84)	4.22**	12.10 (7.51)	0.12
	Externalizing problems	46.13 (9.87)	48.36 (7.82)	− 1.97	7.57 (5.71)	0.472**
	Total problems	50.13 (9.70)	47.07 (9.16)	2.37*	9.18 (5.98)	0.373**
	Affective problems	55.13 (11.81)	52.25 (4.04)	1.96	7.27 (10.00)	0.112
	Anxiety problems	55.61 (7.25)	54.00 (6.31)	2.37	6.67 (6.64)	0.061
	Somatic problems	56.55 (12.78)	51.03 (3.83)	3.50**	9.10 (10.67)	0.111
	ADHD problems	51.72 (7.15)	52.79 (4.95)	− 1.22	3.22 (6.52)	0.336**
	Oppositional defiant	51.78 (10.06)	52.79 (5.37)	− 0.88	4.23 (8.53)	0.37**
SSIS	Communication	16.57 (2.57)	16.68 (3.74)	− 0.21	3.15 (2.62)	0.190
	Cooperation	13.57 (2.64)	14.45 (3.49)	− 2.03*	2.75 (2.27)	0.384**
	Responsibility	13.77 (2.77)	14.63 (3.12)	− 2.08*	2.68 (2.16)	0.371**
	Empathy	13.63 (3.08)	12.54 (3.84)	2.13*	3.06 (2.95)	0.305*
	Engagement	14.11 (3.88)	14.58 (4.72)	− 1.04	4.32 (3.20)	0.165
	Autism	7.94 (4.95)	6.94 (5.60)	1.22	5.23 (4.10)	0.227
	Social skills SS	98.66 (12.50)	102.83 (14.32)	− 2.03*	13.31 (10.58)	0.244
	Problem behaviors SS	97.72 (11.51)	93.89 (9.84)	2.44*	9.89 (8.70)	0.305**
PedsQL	Total emotional	77.65 (18.91)	69.94 (20.53)	2.95**	20.12 (14.77)	0.272*
	Total social	78.19 (19.15)	77.41 (22.38)	0.33	16.92 (13.57)	0.460**
	Total psychosocial	75.09 (15.73)	71.61 (17.18)	1.81	14.25 (10.65)	0.461**
PROMIS	Social relationships	49.86 (10.71)	46.65 (10.47)	2.14*	10.59 (8.02)	0.253*

*Note.* *p < .05; ** *p* < .01

*SSIS*: Only the Cooperation, Responsibility, Empathy, and Problem Behaviors Standard scales yielded significant inter-rater correlations between parents and teachers (*p’s* < 0.05). Parents had significantly higher ratings on the Empathy and Problem Behaviors Standard scales (*p’s* < 0.05); however, teachers had higher ratings on the Cooperation, Responsibility, and Social Skills Standard scale (*p* < 0.05). Magnitudes between ratings (|parent–teacher|) ranged from Responsibility (2.68 ± 2.16) to Social Skills Standard (13.31 ± 10.58).

*PedsQL:* Significant inter-rater correlations between parents and children were found on all three PedsQL scales: Emotional (*p* < 0.05), Social (*p* < 0.01), and Psychosocial (*p* < 0.01). Parents reported significantly higher ratings on the Emotional functioning scale (*p* < 0.01). Mean magnitudes (|parent–child|) between ratings on the Emotional, Social, and Psychosocial scales were 20.12 ± 14.77, 16.93 ± 13.57, and 14.25 ± 10.65, respectively.

*PROMIS:* Parents and child self-report ratings on the PROMIS were significantly correlated (*p* < 0.05); however, the mean scores of parent’s ratings were higher (*p* < 0.05). The average distance (|parent–child|) between parent and child scores was 10.59 ± 8.02.

### Quantifying % Disagreement

*CBCL/TRF*: On the *CBCL/TRF*, 10% of parent–teacher cases resulted in significant RCI-derived disagreement (distance $\ge$ 23) on the Internalizing Problems scale, 27% of cases disagreed (distance $\ge$ 10) on the Externalizing Problems scale, and 30% of cases disagreed (distance $\ge$ 13) on the Total Problems scale. Given the clinical descriptors noted, clinical % disagreement on the Internalizing, Externalizing, and Total problem scales were 21%, 6%, and 7%, respectively. Across the subscales, RCI-derived percent disagreements ranged from 4% (Somatic) to 21% (Social) and clinical percent disagreements ranged from 3% (Oppositional Defiant) to 27% (Somatic) (see Table 3).

**Table 3:** Percent of rating pairs in significant RCI-derived and clinical disagreement

Measure/subscale		RCI-derived		Clinical	
		Sig diff point	% Disagree	Clinical descriptor grouping	% Disagree
CBCL/TRF	Anxious/depressed	17	7	Borderline clinical/clinical vs normal	13
	Withdrawn/depressed	17	9		13
	Social problems	9	21		7
	Attention problems	5	19		9
	Internalizing problems	23	10		21
	Externalizing problems	10	27		6
	Total problems	13	30		7
	Affective problems	22	7		16
	Anxiety problems	17	9		24
	Somatic problems	25	4		27
	ADHD problems	9	12		4
	Oppositional defiant	12	7		3
SSIS	Communication	7	6	Below average vs average/above average	11
	Cooperation	5	26		15
	Responsibility	5	14		11
	Empathy	6	15		17
	Engagement	9	12		35
	Autism	10	15		25
	Social skills SS	24	14		18
	Problem behavior SS	17	20		14
PedsQL	Emotional functioning	33	17		
	Social functioning	21	29		
	Psychosocial functioning	18	35		
PROMIS	Social relationships	18	18	Poor vs fair/good/excellent	5

*SSIS:* The *SSIS* resulted in 14% parent–teacher RCI-derived disagreement (distance $\ge$ 24) and 18% clinical disagreement on the Social Skills Standard and 20% RCI-derived disagreement (distance $\ge$ 17) and 14% clinical disagreement on the Problem Behavior Standard. Across the subscales, RCI-derived percent disagreements ranged from 6% (Communication) to 26% (Cooperation), and clinical percent disagreements ranged from 11% (Communication and Responsibility) to 35% (Engagement).

*PedsQL & PROMIS:* The *PedsQL* yielded 17% parent–child disagreement (distance $\ge$ 33) on emotional functioning, 29% disagreement (distance $\ge$ 21) on Social Functioning, and 35% disagreement (distance $\ge$ 18) on Psychosocial Functioning. The *PROMIS* resulted in 18% of parent–child rating pairs in significant RCI-derived disagreement (distance $\ge$ 18), with 5% in clinical disagreement.

## Discussion

Understanding patterns of multi-informant agreement during psychological assessments of childhood cancer populations will inform care for this vulnerable group and offer insight into the collaboration between schools and families during school reintegration. Results from this study indicated low-moderate levels of inter-rater agreement for this population, highlighted through poor-to-moderate ICC values, significant mean rater differences, large magnitudes of inter-informant discrepancy, and high occurrences of disagreements. Perceptions of psychosocial functioning across measures were distinct across informants, with parents perceiving higher levels of psychosocial and behavior problems compared to teachers, and survivors endorsing more psychosocial problems compared to parent reports. Findings from this study add to the literature on inter-rater agreement in chronically ill and childhood cancer populations and underscore the necessity for integrating multi-informant reports of psychosocial functioning in assessment and increasing collaborative approaches between parents, teachers, and children.

Agreement levels between parents and teachers on reports of behavioral and psychosocial functioning for PBTS and PSTS seemed consistent with, if not lower than, previously observed agreement among typically developing and other clinical populations (Achenbach & Rescorla, 2001; Ende & Verhulst, 2005; Gresham et al., 2010; Renk, 2004). Specifically, the ASEBA manual reports significant correlations between CBCL and TRF scores on all subscales (excluding somatic complaints) and reports a mean inter-rater ICC of 0.93. However, results from this study found no significant associations on half the analyzed CBCL/TRF subscales and a mean inter-rater ICC of 0.33. In accordance with prior literature, parent-teacher agreement was higher for externalizing symptoms, such as attention problems, and lower for internalizing symptoms, indicating better observability of externalizing behaviors (De Los Reyes & Kazdin, 2005; Salbach-Andrae et al., 2009). Parent-teacher agreement levels on social functioning on the SSIS were also in line with previous studies, indicating trends of poor-to-moderate levels of social skills agreement (De Los Reyes & Kazdin, 2005; Gresham et al., 2010).

It is important to consider the potential role of school absenteeism on these lower levels of parent–teacher agreement, as survivors were only a mean of 3.4 months from treatment at the time of assessment and missed a median of 2–5 months of school. In addition, increased parent perceptions of child vulnerability due to cancer experiences may partially contribute to the significantly poorer parent ratings of psychosocial functioning compared to teachers observed in this study (e.g., CBCL internalizing problems, SSIS social skills) (Staba Hogan et al., 2018). Results highlight critical gaps, specifically in internalizing problems and social functioning, across parent–teacher mutual understanding of survivor psychosocial functioning in the post-treatment period. These gaps in mutual understanding of the survivors functioning are consistent with the current literature highlighting significant barriers in clinician-parent-teacher collaboration such as insufficient and poorly timed communication and poor education on potential neurocognitive and psychosocial late effects (Parrillo et al., 2022). As clinician–family–school collaborations are paramount to child success post-treatment, results of this study underscore the necessity for increased ongoing education and communication between parties to ensure mutual understanding of child functioning and necessary intervention strategies across contexts (Thompson et al., 2015).

Poor-to-moderate levels of agreement were also found on parent–child reports of psychosocial functioning, consistent with the current agreement literature in cancer populations; however, the directionalities of agreement were distinct (Varni et al., 2002). While a past study has found that parents of children in cancer or chronically ill groups tend to report lower levels of functioning compared to self-report, parents of PBTS and PSTS in this study had significantly higher ratings of healthy emotional functioning on the PedsQL and peer relationship quality on the PROMIS than that of the child reports (Parsons et al., 1999). Differences in these parent–child rating directionalities across investigations may be influenced by differences in the QoL measures and populations assessed, as Parsons’ study assessed QoL in bone marrow transplant survivors specifically, using the Child Health Rating Inventories (general & disease specific). Agreement patterns between parents and children may be diagnosis-specific, as differences in treatment experiences could lead to varying psychosocial functioning and perception abilities. In addition, the timing of assessment from treatment could impact the directionality of ratings. As children in this study were more recently reintegrated into school, self-comparisons with other children in class could lead to the lower personal reports of emotional functioning and peer relationship quality. Thus, the influence of diagnosis, treatment method and timing, and assessment method on parent/self-perception and parent–child agreement of QoL should be further examined across additional pediatric cancer survivor groups.

Previous research highlighting the importance of parental understanding of child functioning on family engagement in clinical support underscores the significance of addressing this gap in parent–child communication, as parents are the primary advocates for children (Morrissey-Kane & Prinz, 1999). Clinicians should seek to foster a family environment that encourages open conversations about psychosocial functioning to address these differences in shared understanding. Additionally, the poor levels of agreement and large discrepancies on scales signify the importance of integrating self-report measures of functioning in the clinical process instead of solely relying on parent report.

Given the prevalence of low-to-moderate agreement patterns between parent–child–teacher reports of child functioning, future work should investigate procedures for integrating multiple informant perspectives and predictors of inter-informant agreement. As outlined in JCCAP’s 2023 special issue on informant discrepancies, future research constructing evidence-based approaches for integrating multi-informant reports without diminishing any informant’s perspective on child functioning is critically needed (De Los Reyes & Epkins, 2023). In addition, factors such as diagnosis, treatment, stress, child and family functioning, socioeconomics, and school absenteeism should be evaluated as predictors of agreement specific to childhood cancer. Understanding these predictors offers insight into the underlying mechanisms of parent–child–teacher dynamics and informs clinical assessments and collaboration during the post-treatment period.

Strengths of this study include the use of three informant perspectives across home and school contexts and a comprehensive subscale analysis of psychosocial functioning across multiple measures. However, study findings should be interpreted within the context of its limitations. Agreement was assessed cross-sectionally at one time point, in the immediate post-treatment period (mean 3.4 months off treatment), which may limit the generalizability of agreement patterns to other cancer populations further from treatment. As brain and non-CNS solid tumor groups were combined for this analysis but may have varying psychosocial profiles and self-assessment abilities, the findings of the study are limited to broader pediatric populations since the potential influence of diagnostic group on agreement levels were not assessed. In addition, the level of post-treatment collaboration between families and schools was not measured, which could have offered further insight into the efficacy of support services.

Among the PBTS and PSTS populations, parents, teachers, and children each have distinct perspectives of the child that must be integrated and communicated for collaborative intervention approaches. Future work should further analyze the underlying mechanisms of agreement and procedures for integrating ratings to optimize collaborative intervention approaches. Addressing these identified differences in understanding of the child’s psychosocial functioning will ultimately improve outcomes for pediatric cancer survivors in the post-treatment school-reintegration period.

## Supplementary Information

Below is the link to the electronic supplementary material.

Supplementary file1 (DOCX 21 kb)

## Data Availability

The data that support the findings of this study are available upon request from the corresponding author. The data are not publicly available due to their containing information that could compromise the privacy of the research participants.
