# Supplementary material for: Inter-rater Agreement in Multi-informant Reports of Psychosocial Functioning of Pediatric Brain and Solid Tumor Survivors
Source: J Clin Psychol Med Settings. 2024 Dec 4;32(2):297–305. doi: 10.1007/s10880-024-10059-9 (PMC12081537; doi:10.1007/s10880-024-10059-9)
Supplement: Supplementary file 1 — Supplementary file1 (DOCX 21 kb) [file 10880_2024_10059_MOESM1_ESM.docx]

# **Appendix**

**Supplemental Table 1** Internal Consistencies and Sample Items for each Measure

| **Measure/Subscale** | | **Internal Consistency (alpha) (Parent)** | **Internal Consistency (alpha) (Teacher/Self)** | **Sample Item(s)** |
| --- | --- | --- | --- | --- |
| **CBCL/TRF** | Anxious/Depressed | 0.796 | 0.722 | Fears certain animals, situations, or places, other than school |
|  | Withdrawn/Depressed | 0.756 | 0.78 | There is very little he/she enjoys |
|  | Social Prob | 0.755 | 0.706 | Complains of loneliness |
|  | Attention Prob | 0.837 | 0.908 | Can’t concentrate, can't pay attention for long |
|  | Internalizing Prob | 0.862 | 0.795 |  |
|  | Externalizing Prob | 0.863 | 0.92 |  |
|  | Total Prob | 0.924 | 0.962 |  |
|  | Affective Prob | 0.691 | 0.706 | Underactive, slow moving, or lacks energy |
|  | Anxiety Prob | 0.705 | 0.68 | Nervous, high-strung, or tense |
|  | Somatic Prob | 0.676 | 0.715 | Physical problems without known medical cause: headaches, rashes, vomiting, etc. |
|  | ADHD Prob | 0.769 | 0.846 | Inattentive or easily distracted |
|  | Oppositional Defiant | 0.743 | 0.792 | Argues a lot |
|  | Conduct Prob | 0.527 | 0.822 | Cruelty, bullying, or meanness to others |
| **SSIS** | Communication | 0.733 | 0.85 | Takes turns in conversations |
|  | Cooperation | 0.828 | 0.894 | Follows your directions |
|  | Assertion | 0.264 | 0.77 | Expresses feelings when wronged |
|  | Responsibility | 0.864 | 0.807 | Is well-behaved when unsupervised |
|  | Empathy | 0.874 | 0.883 | Feels bad when others are sad |
|  | Engagement | 0.85 | 0.886 | Participates in games or group activities |
|  | Autism raw | 0.811 | 0.848 | Uses odd physical gestures in interactions |
|  | Social Skills SS | 0.948 | 0.959 |  |
|  | Problem Behaviors SS | 0.881 | 0.894 |  |
| **PedsQL** | Total Emotional | 0.811 | 0.721 | Worrying about what will happen, Feeling afraid or scared |
|  | Total Social | 0.774 | 0.827 | Getting along with other children, Keeping up when playing with other children |
|  | Total Psychosocial | 0.876 | 0.874 |  |
| **PROMIS** | Social Relationships | 0.926 | 0.898 | Feeling accepted by other kids, Good at making friends |

**Supplemental Table 2** ICC values using a two-way mixed, absolute agreement model

| **Measure/Subscale** | | **ICC** | **Lower C.I** | **Upper C.I** |
| --- | --- | --- | --- | --- |
| **CBCL** | Anxious/Depressed | 0.03 | -0.547 | 0.400 |
|  | Withdrawn/Depressed | 0.11 | -0.341 | 0.422 |
|  | Social Problems | 0.49 | 0.180 | 0.681 |
|  | Attention Problems | 0.71 | 0.535 | 0.823 |
|  | Internalizing Problems | 0.18 | -0.220 | 0.461 |
|  | Externalizing Problems | 0.62 | 0.386 | 0.765 |
|  | Total Problems | 0.53 | 0.239 | 0.706 |
|  | Affective Problems | 0.12 | -0.392 | 0.454 |
|  | Anxiety Problems | 0.11 | -0.430 | 0.452 |
|  | Somatic Problems | 0.10 | -0.351 | 0.417 |
|  | ADHD Problems | 0.48 | 0.152 | 0.677 |
|  | Oppositional Defiant | 0.47 | 0.140 | 0.675 |
| **SSIS** | Communication | 0.31 | -0.148 | 0.578 |
|  | Cooperation | 0.53 | 0.236 | 0.710 |
|  | Responsibility | 0.53 | 0.233 | 0.708 |
|  | Empathy | 0.45 | 0.108 | 0.658 |
|  | Engagement | 0.28 | -0.176 | 0.561 |
|  | Autism | 0.37 | -0.038 | 0.614 |
|  | Social Skills SS | 0.38 | 0.000 | 0.616 |
|  | Problem Behavior SS | 0.45 | 0.110 | 0.657 |
| **PedsQL** | Emotional Functioning | 0.41 | 0.098 | 0.610 |
|  | Social Functioning | 0.63 | 0.423 | 0.759 |
|  | Psychosocial Functioning | 0.62 | 0.417 | 0.754 |
| **PROMIS** | Social Relationships | 0.39 | 0.051 | 0.613 |
